# Epigallocatechin-3-Gallate (EGCG)-Loaded Hyaluronic Acid Hydrogel Seems to Be Effective in a Rat Model of Collagenase-Induced Achilles Tendinopathy

**DOI:** 10.3390/jfb16020055

**Published:** 2025-02-10

**Authors:** Hwa Jun Kang, Sivakumar Allur Subramanian, Si Young Song, Jihyun Hwang, Collin Lee, Sung Jae Kim

**Affiliations:** 1 Department of Orthopedic Surgery, Dongtan Sacred Hospital, Hallym University College of Medicine, Hwaseoung 18450, Republic of Korea; ospigy@naver.com (H.J.K.); drsivaphdbio@gmail.com (S.A.S.); superdrsys@hotmail.com (S.Y.S.); 2 Department of Biomedical Engineering, Johns Hopkins University School of Medicine, Baltimore, MD 21205, USA; jhwang50@jh.edu; 3 Department of Biology, University of Maryland—College Park, College Park, MD 20742, USA; clee1241@terpmail.umd.edu

**Keywords:** Achilles tendinopathy, Epigallocatechin-3-gallate, hyaluronic acid, reactive oxygen species

## Abstract

Tendon injuries account for 45% of musculoskeletal injuries. However, research on the occurrence and pathogenesis of tendinopathy is insufficient, and there is still much debate regarding treatment methods. It is important to understand the molecular mechanisms of oxidative stress and inflammatory responses because oxidative stress in tendon tissue is induced by various factors, including inflammatory cytokines, drug exposure, and metabolic abnormalities. In this study, 28 rats were divided into four groups (7 rats assigned to each group): control group (CON), collagenase injection group (CL), collagenase injection and hyaluronic acid injection group (CL + HA), and collagenase injection and EGCG-loaded hyaluronic acid injection group (CL + HA + EGCG). Seven weeks after the start of the study, all rats underwent histochemical analysis, immunofluorescence staining, and Western blot. The results showed increased inflammatory cells, disarray of collagen matrix, and degradation of the collagen matrix in the CL group. However, in the EGCG-treated group, there was a significant increase in type I collagen expression and a significant decrease in type III collagen expression, compared to the CL group. Additionally, there was an increase in the expression of antioxidant markers SOD (Superoxide Dismutase) and CAT (Catalase), tenogenic markers COLL-1 (collagen type I), and SCX (Scleraxis), and a downregulated expression of apoptosis markers cas-3 and cas-7. Our findings suggest that EGCG-loaded hyaluronic acid hydrogel exhibits potential in preventing tendon damage and promoting the regeneration process in a rat model of Achilles tendinopathy. The insights gained from our histological and molecular investigations highlight the future potential for testing novel tendinopathy treatments in human subjects.

## 1. Introduction

Tendon injuries, such as Achilles, patellar, and supraspinatus tendinopathy, are common in athletes and middle-aged individuals, playing a significant role in orthopedic treatment [1]. The severity of tendon injuries ranges from temporary pain to chronic inflammation, partial tears, or full-thickness ruptures [1,2]. The healing of damaged tendons is related to the impaired metabolic activities of resident cells that determine the homeostasis of the injured tissue [3,4]. In histopathology analysis, tendon injuries may exhibit collagen degeneration, fiber disintegration, and increased vascularization [5,6]. Despite their clinical importance, research on the occurrence and pathogenesis of tendinopathy is insufficient, and advancements in treatment remain limited [7].

The inflammatory response in tendinopathy is characterized by the presence of proinflammatory cytokines as well as matrix metalloproteinases (MMPs). As a result, the cells that form tendon tissues are damaged, leading to the deterioration of tissue integrity. Therefore, various pharmacological treatments that suppress the inflammatory response, such as non-steroidal anti-inflammatory drugs (NSAIDs) and corticosteroid injections, are used [8,9]. However, prolonged use of NSAIDs can lead to elevated liver enzyme levels and impaired kidney function, while adverse drug interactions may occur when used in combination with other medications [10,11]. Furthermore, several cohort studies report that high doses of corticosteroid injections reduce pain and improve tendon function, while other reports indicate no difference in pain relief effects [12]. Long-term steroid injections can induce cell aging, toxicity, and inflammation in damaged tendon cells, with serious side effects such as tendon rupture [13,14]. Thus, current conservative treatments remain popular but are limited [15]. Recent advancements in technology identified future alternative treatments, including biomaterials, extracellular vesicles, and exosome-loaded hydrogels such as hyaluronic acid (HA) [16,17,18,19]. In cases where there is no improvement despite prolonged non-surgical treatment, surgical treatment may be considered [20,21]. However, surgical treatments do not have high success rates and involve long recovery periods, leading to ongoing debate about the optimal treatment method [22].

Tissues sensitive to physical stress, such as tendons, are continuously exposed to oxidative stress during exercise. Reactive oxygen species (ROS) can damage lipids, proteins, and DNA in cells and tissues, leading to tendon fibrosis, adhesions, and pathological changes associated with tendinopathy. However, the exact relationship with oxidative stress is not well understood. The action of antioxidants is known to prevent ROS formation and promote collagen biosynthesis, thereby protecting tendon cells from oxidative stress. Given these findings, epigallocatechin gallate (EGCG) is known as an antioxidant supplement that helps reduce the adverse effects of H_2_O_2_-mediated cytotoxicity [23]. Feng et al. [24] reported that EGCG can reduce pain and suppress the expression of proinflammatory genes in patients with rotator cuff tendinopathy. EGCG is a polyphenolic compound consisting of an ester formed between epigallocatechin and gallic acid, giving a structure with multiple hydroxyl groups that contribute to strong antioxidant activity. As the most abundant catechin in green tea, it plays a key role in protecting cells from oxidative stress and regulating inflammatory pathways. EGCG has been widely studied for its potential therapeutic applications in various fields of medicine [23,24].

Additionally, in synovial fibroblasts of rheumatoid arthritis, EGCG has been reported to inhibit the synthesis of matrix metalloproteinase-1 (MMP-1) and MMP-3 induced by TNF-alpha [25]. Hsiao et al. [26] found that encapsulated EGCG hydrogels protect tendons by regulating the expression of type I/III collagen in a tendinopathy model These various studies suggest that EGCG has significant antioxidant properties.

In this study, we analyzed the therapeutic effects of EGCG loaded within hydrogel in a rat model of Achilles tendinopathy induced by collagenase.

## 2. Materials and Methods

### 2.1. Preliminary Study: Determination of Collagenase Dosage and Timing for Inducing Tendinopathy in the Rat Achilles Tendon (In Vivo)

#### 2.1.1. Experimental Animals

Seven male Sprague Dawley rats (10 weeks old, 250 ± 5 g body weight) were used in this study. The rats were housed individually in polypropylene cages under hygienic conditions at a temperature of 22–24 °C with a 12 h light/12 h dark cycle. Animals were provided ad libitum access to water and standard pellet feed. The rats were randomly divided into seven groups (Table 1), which were used to determine the appropriate collagenase injection dosage for inducing tendinopathy in the rat Achilles tendon.

#### 2.1.2. Surgical Procedure and Sample Collection

**Figure 1:**
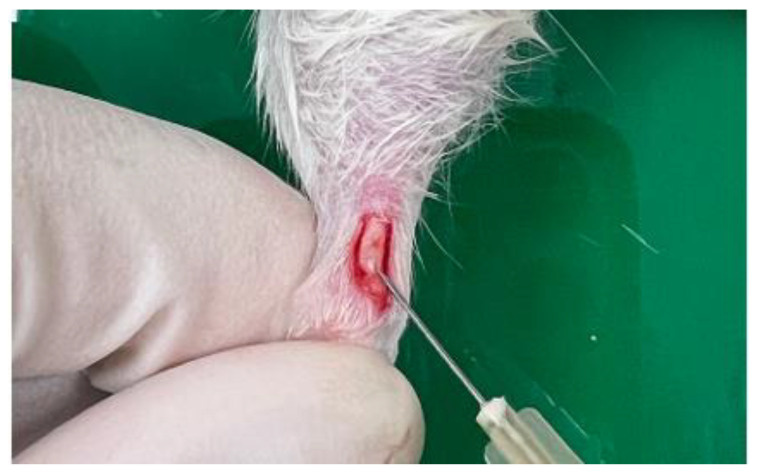
Procedure for exposing the central region of the Achilles tendon in experimental rats and injecting type I collagenase into the central tendon region.

Rats were anesthetized with 3% isoflurane (Merial, Duluth, GA, USA) via inhalation. The left hind limb of each rat was shaved and disinfected, and a 1 cm longitudinal skin incision was made via a central approach to expose the mid-region of the Achilles tendon through blunt dissection. Type I collagenase dissolved in 0.9% PBS was injected into the central portion of the tendon using a 30-gauge needle, with different doses allocated to each group (Figure 1). A single orthopedic surgeon performed all procedures using consistent methods for all animals. The skin was sutured using Vicryl Rapide™ 4-0 sutures (Johnson & Johnson, New Brunswick, NJ, USA) with separate knots. To facilitate recovery from systemic anesthesia, atipamezole (1 mg/kg; Antisedan, Pfizer, New York City, NY, USA) was administered subcutaneously. Achilles tendons were harvested at 3, 14, and 18 days post-injection under isoflurane anesthesia for subsequent histological analysis.

### 2.2. Histological Analysis: Hematoxylin and Eosin (H&E), Masson’s Trichrome (MT), and Picrosirius Red (PR) Staining

Tendon samples were fixed in 10% formalin for 24 h, dehydrated using an ethanol gradient, embedded in paraffin, and sectioned longitudinally into 5 μm slices. The slides were stained with H&E, MT, and PR to evaluate the structural changes in the tendons injected with collagenase compared to untreated normal tendons. Microscopic images were captured using an Olympus IX71 optical microscope and an Olympus IX73 camera (Olympus, Tokyo, Japan). Histological evaluation included analysis of fiber structure and arrangement, resident cell density and morphology, inflammatory cell infiltration, neovascularization, and lipid deposition. Based on this scoring system, normal tendons were assigned a score of 0, while the maximum score for abnormal tendons was 18. A collagenase concentration of 3 mg/mL (30 μL) produced the most prominent features of tendinopathy in terms of tissue damage, as shown in the data provided in the results. This dosage was determined to be reliable and optimal for establishing the tendinopathy model in rats for further studies.

### 2.3. Main Experiment: Analysis of the Beneficial Effects of Cross-Linked Hyaluronic Acid Hydrogel Injection (In Vivo)

#### 2.3.1. Surgical Procedures and Sample Collection

**Figure 2:**
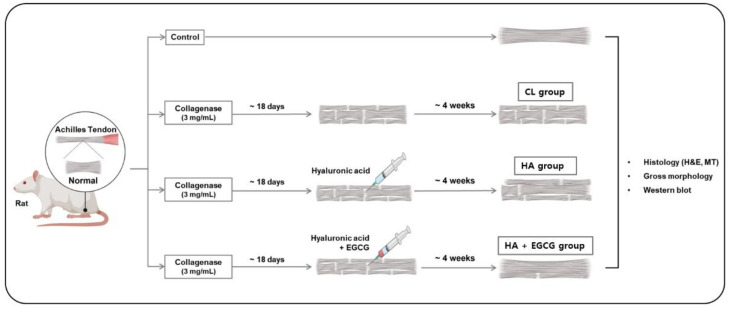
Schematic abstract of study design.

A total of 28 male Sprague Dawley rats, weighing between 250 g and 260 g, were used for this study (n = 7). During our pilot study to model degenerative Achilles tendinopathy in a rat model, we found that 30 μL of collagenase (3 mg/mL) at day 18 following collagenase injection best mimicked degenerative tendinopathy. Therefore, we standardized this dosage of collagenase for the entire study. Eighteen days after the collagenase injection, a 2 cm incision was made at the injection site. Under direct visualization, 30 μL of EGCG (10 mg/kg b.w/rat (62.5 mg/mL) mixed with 1 mL of HA) and HA alone were injected at the same site for each respective group. The skin was sutured as previously described. We used an intraperitoneal injection of diclofenac (1 mg/kg) on the day of surgery for pain control. Four weeks after treatment, the rats were euthanized using an overdose of CO_2_, and Achilles’ tendons were harvested for evaluation (n = 7 tendons per time point per group). Each tendon was cut into 2 samples for histological analysis and Western blot. whole-study designs are depicted in Figure 2. The ethical approval for this study was obtained from IRB/EC of Dongtan Sacred Heart Hospital [HMC 2022-5-1018-50].

#### 2.3.2. Hematoxylin and Eosin (H&E) and Masson’s Trichrome (MT) Staining

Slides of rats’ Achilles’ tendons were prepared. In brief, the Achilles tendon was washed in PBS, frozen in cooled hexane, and freeze-embedded with 4% to 5% carboxymethyl cellulose in the coolant. After a specially prepared adhesive film was fastened to the cut surface, the sample was cut longitudinally into 5 mm-thick sections. The sections were stained with hematoxylin–eosin (H&E) and Alcian blue, and then mounted onto 3-amino-coated slides. The specimens were examined by standard light microscopy, and photomicrographs were obtained at 200× magnifications.

#### 2.3.3. Western Blot Analysis

The Achilles tendon from each left hind limb was removed. Proteins were extracted from 100 mg of tendon samples and homogenized in RIPA lysis buffer supplemented with protease inhibitors. Protein concentrations were measured with the Pierce BCA Protein Assay Kit (Thermo Scientific^TM^, Waltham, MA, USA). Extract samples containing 30 μg of protein were solubilized in Laemmli buffer, separated by SDS-PAGE gel and then transferred to PVDF membranes (GE Healthcare Inc., Amersham, UK) for 1 h. These PVDF membranes were blocked with 5% skimmed milk powder in 0.5 M Tris-buffered saline (pH 7.4) with 0.05% Tween 20 (TBST) at room temperature for 2 h. The membrane was incubated overnight at 4 °C with primary antibodies SOD (1:1000), CAT (1:1000), COLL-1 (1:1000), COLL-3 (1:1000), NF-κB (1:1000), and GAPDH (1:1000). After washing three times with TBST, these membranes were probed with HRP-conjugated secondary antibodies (1:5000 dilutions) for 60 min at room temperature and then washed three times with TBST (10 min per wash). Protein bands were observed using an enhanced chemiluminescence assay kit from Thermo Scientific. Bands were imaged using the Amersham Imager 600 (GE Healthcare Life Sciences, Little Chalfont, UK) and quantified using ImageJ (Version 1.53, National Institutes of Health, Bethesda, MD, USA) software. The results are expressed as density ratio to GAPDH after normalization. SOD was analyzed to assess the antioxidant capacity in the regenerating tendons. This enzyme is critical for the dismutation of superoxide radicals into hydrogen peroxide, thereby protecting cells from degeneration-induced oxidative damage. CAT expression was measured to evaluate the breakdown of hydrogen peroxide into water and oxygen. This enzyme is key in mitigating oxidative stress, a known contributor to tendon degeneration. COLL-1 was used to quantify the primary structural protein of the tendon extracellular matrix (ECM). Its presence indicates the extent of tendon regeneration and the restoration of mechanical integrity. COLL-3 expression was analyzed as it is associated with early stages of tendon repair and tissue remodeling. Scx, a transcription factor specific to tendon fibroblasts, was assessed as a marker of tendon differentiation and repair. Its expression reflects the activation of tendon-specific cellular pathways during regeneration. Cas-3 was measured to detect apoptosis, as this effector caspase is involved in the execution phase of programmed cell death. Cas-7 expression was examined to further explore apoptotic pathways.

#### 2.3.4. Statistical Analysis

All experiments were repeated at least three times (n = 3), and the results were represented as Mean ± Standard deviations. One-way analysis of variance (ANOVA) was used for statistical evaluation, followed by Duncan’s multiple range test (DMRT). A *p*-value of ≤0.05 was considered statistically significant.

## 3. Results

### 3.1. Establishment of a Chronic Tendinopathy Model Through Preliminary Experiments

**Figure 3:**
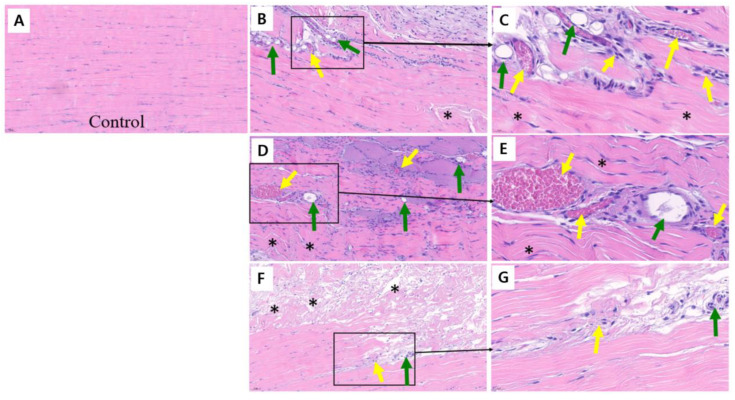
Preliminary experiments of histological evaluation of collagenase-induced chronic tendinopathy in rat Achilles tendons for establishing a chronic tendinopathy model (H&E staining). (**A**) Control tendon showing normal alignment of collagen matrix and minimal cellularity, with no evidence of inflammation or extracellular matrix disruption. (**B**) Tendon treated with collagenase 30 μL (10 mg/mL) at 14 days, displaying disorganized collagen matrix, cellular infiltration (yellow arrows), and regions of extracellular matrix degradation. (**C**) Magnified view of a region from (**B**), highlighting prominent cellular infiltration (yellow arrows) and localized collagen fiber disruption, reflecting acute tendon injury pattern. (**D**) Tendon treated with collagenase 50 μL (10 mg/mL) at 14 days, showing further fiber disorganization, increased cellular infiltration (yellow arrows), and visible neovascularization (green arrows). (**E**) Magnified region from (**D**), emphasizing areas of inflammatory cell aggregation (yellow arrows) and vascular proliferation (green arrows). (**F**) Tendon treated with collagenase 30 μL (10 mg/mL) at 18 days, demonstrating severe collagen matrix disruption, extensive extracellular matrix degradation, and decreased inflammatory cell infiltration (yellow arrows). (**G**) Magnified region from (**F**), illustrating advanced tissue damage, including severe fiber disorganization and dense inflammatory infiltration, reflecting chronic lesion (yellow arrows). *, collagen fiber.

The tendon samples from the group treated with 30 μL of 3 mg/mL collagenase exhibited a severely maligned and disrupted collagen matrix at 18 days post-injection. This was accompanied by extensive degradation of the collagen matrix, indicative of significant extracellular matrix breakdown. Furthermore, the structural integrity of the tendon tissue was profoundly compromised, highlighting key characteristics of chronic tendinopathy (Figure 3).

### 3.2. Sample Collection

**Figure 4:**
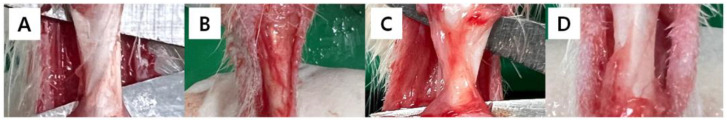
The gross observations at the final tissue collection time point for rats in each group: CON (**A**), CL (**B**), CL + HA (**C**), and CL + HA + EGCG (**D**).

The gross observations of the rat model groups are as follows: In the control group (CON), bright white tendon tissue was observed with no signs of congestion or discoloration in the surrounding tissue, presenting a clean appearance (Figure 2). Conversely, in the collagenase injection with no treatment group (CL), congestion and vascular changes in the surrounding tendon tissue were noted, and upon palpation, there was a decrease in tendon tension. The hyaluronic acid injection group (HA) exhibited increased congestion in the surrounding tissue compared to the control group, but significantly less congestion than the collagenase injection group. In the EGCG-loaded hyaluronic acid injection group (HA + EGCG), there was less congestion in the surrounding tendon tissue compared to both the CL group and the HA group, presenting visual observations most similar to the normal tendon tissue in the control group (Figure 4).

### 3.3. Hematoxylin–Eosin (H&E) and Masson’s Trichrome (MT) Staining

**Figure 5:**
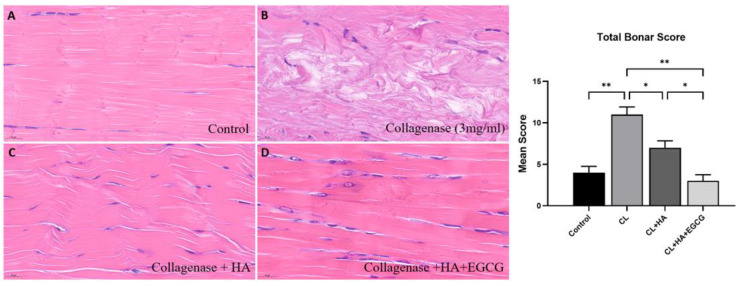
H&E staining showed that the HA +EGCG group exhibited tendon cell (spindle cell) morphology and proliferation, collagen matrix characteristics, collagen matrix alignment maintenance, and vascularity most resembling that of the control group. (**A**) Control group, normal tendon architecture without degradation. (**B**) Collagenase-treated group, severe tissue degradation and disrupted collagen structure. (**C**) Collagenase + HA group, partial restoration of collagen structure, with improved tissue alignment. (**D**) Collagenase + HA + EGCG group, enhanced recovery of collagen structure and alignment compared to the Collagenase + HA group, indicating a synergistic effect of HA and EGCG on tissue repair. Bonar score ranges from 0 to 12, indicating the severity of pathological changes in the tendon. The CL group showed a significant increase in the total Bonar score compared to the control group. Both the HA and HA + EGCG groups exhibited significantly reduced Bonar scores compared to the CL group. * *p* < 0.05; ** *p* < 0.01.

**Figure 6:**
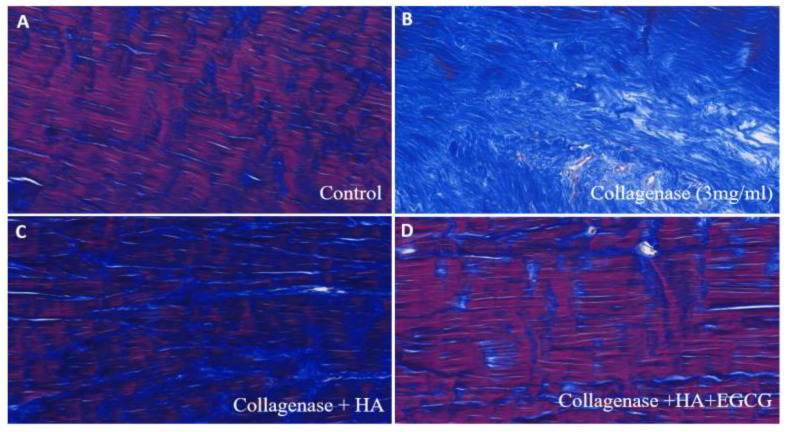
Masson’s trichrome staining at 4 weeks post-treatment; the HA + EGCG-treated group showed the best collagen matrix realignment compared to the other groups. (**A**) Control group, well-organized and intact collagen fibers with minimal disruption. (**B**) Collagenase-treated group, Significant degradation and disorganization of collagen fibers, with marked loss of structural integrity. (**C**) Collagenase + HA group, partial restoration of collagen structure and improved fiber alignment, suggesting the protective effect of HA. (**D**) Collagenase + HA + EGCG group, substantial recovery of collagen fiber organization and enhanced structural integrity compared to the Collagenase + HA group, indicating a synergistic effect of HA and EGCG.

In the H&E-stained samples, the CL group showed collagen fiber degeneration, and the HA+ EGCG group showed a lower level of degenerative changes in tendons compared to the other groups (Figure 5). Moreover, the HA+ EGCG group exhibited improved collagen fiber alignment, cellularity, and hypervascularity. The MT staining revealed that the CON group showed a physiologically normal tendon that appeared uniformly red, while the tendons in CL group were stained completely blue at the level of the core lesion site. The HA + EGCG group showed recovered the collagen fiber alignment (Figure 6).

### 3.4. EGCG Increased SOD and CAT Expression for Antioxidant and Anti-Inflammation

**Figure 7:**
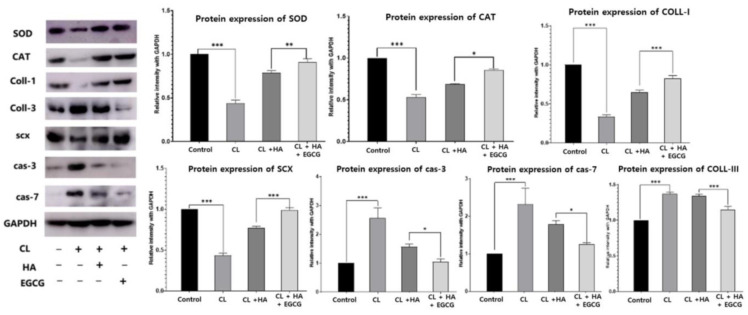
Western blot analysis was used to investigate the protein expression of SOD, CAT, COLL-1, COLL-III, SCX, Cas-3, and Cas-7 in the experiment groups. * *p* < 0.01, ** *p* < 0.001, *** *p* < 0.0001.

The indicators that illustrate the intensity of proteins related to antioxidant and oxidative stress, such as SOD and CAT; tenogenic markers such as COLL-1 and III, SCX; and apoptotic markers such as cas-3 and cas-7 were determined by Western blot (Figure 7). In the evaluation of antioxidant effects using Western blot analysis, the HA and HA + EGCG groups showed an increased expression of SOD and CAT compared to the CL group (*p* < 0.0001, for both). Furthermore, the HA + EGCG group exhibited a significantly higher expression of these markers compared to the HA group. Regarding markers of tendon cell differentiation and ECM synthesis, the treatment groups demonstrated an increased expression of COLL-I and SCX proteins relative to the CL group (*p* < 0.0001, for both). Notably, the HA + EGCG group showed a significantly enhanced expression of these proteins compared to the HA group. In the assessment of apoptotic activity, the treatment groups exhibited reduced expressions of apoptotic markers compared to the CL group (*p* < 0.0001), with the HA + EGCG group showing a significantly greater suppression of apoptotic marker expression than the HA group (*p* < 0.01).

## 4. Discussion

The treatment with hyaluronic acid (HA) hydrogels containing EGCG seemed to effectively activate tenocytes to increase collagen matrix synthesis and prevent tenocyte apoptosis with its antioxidative effect. Increased expression of collagen type I and reduced expression of collagen type III imply that the HA + EGCG treatment also has a tendon ECM remodeling effect.

Approximately 90% of the collagen in normal tendons is type I, whereas type III collagen is upregulated during inflammation [27]. The abnormal collagen ratio (i.e., a high ratio of type III to type I collagen) manifests in the degenerative tendon [4]. Li et al. [28] reported that the polyphenol compound eugenol enhances the production of COLL-I and COLL-III, regulating tendon matrix formation and providing an environment to maintain a stretch for tendon cells. Our data confirm that EGCG can also help regulate the COLL-I and COLL-III ratio during tendinopathy. Therefore, rapid replacement of type III collagen into type I collagen is necessary for tendon repair [4].

Oxidative stress at tendon injury sites has been identified as a major factor contributing to degenerative events and the disruption of ECM organization in tendons [25,29]. Moreover, oxidative stress-induced damage of tendon stem cells presents a potential therapeutic target for tendon repair [28,30]. Natural polyphenolic compounds, such as quercetin, possess strong antioxidative properties that might be effective against collagenase tendinopathy by inhibiting oxidative stress through the activation of SOD, CAT, and Gpx [31]. Our data show that EGCG promotes the activity of SOD and CAT by inhibiting oxidative stress and its antioxidant properties [24]. Additionally, the inhibition of oxidative stress by EGCG suggests its potential application in diabetic bone models, where protection against diabetes-induced oxidative damage may improve osteoblast and ECM integrity [32].

Additionally, EGCG treatment effectively promoted the protein expression of tendon formation markers such as SCX. SCX is a tenogenic differentiation factor that positively regulates COLL-I and tenomodulin [33]. A recent study demonstrated that TGF-β1 can stimulate tenogenesis in bone marrow-derived mesenchymal stem cells by increasing the transcription factor SCX [34].

A previous study reported that tendon damage leads to degenerative changes by increasing caspases in tendons, thereby reducing their functional ability [35]. Quercetin was observed to attenuate apoptosis by suppressing cas-3 expression. It is thought that the anti-apoptotic property of quercetin in tendinopathy prevents the formation of free radicals owing to its antioxidant structure, which provides protection against apoptosis [31]. Analogously, HA + EGCG could prevent apoptosis and promote cellular action in tendinopathy, as demonstrated in the present study. Hsiao et al. developed a sustained-release regimen using an oxidized hyaluronic acid/adipic acid dihydrazide hydrogel as both a drug carrier and therapeutic agent for tendinopathy treatment. Their study demonstrated that incorporating EGCG into this hydrogel conferred additional protective effects in both in vitro and in vivo tendinopathy models, suggesting that EGCG’s anti-inflammatory and antioxidative properties may mitigate tendinopathy progression [26]. Ahmed et al. reviewed the efficacy of EGCG in protecting cartilage from degradation in joint disorders. The findings provide a scientific rationale for the efficacy of EGCG in protecting cartilage breakdown during the progress of joint disorders and could be utilized in other chronic ailments where the integrity of collagen is compromised in tissue destruction or remodeling [36]. Collectively, these studies suggest that EGCG’s anti-inflammatory and antioxidative properties may offer therapeutic benefits in managing tendinopathy. Our findings align with this body of research, further supporting the potential of EGCG as a treatment modality for tendinopathy.

The current study has certain limitations. This study lacks a vehicle-treated sham group, which would have provided a more robust baseline for comparison. Future studies should aim to include this control to better isolate the effects of the EGCG-loaded hyaluronic acid hydrogel. Another limitation of this study is the absence of a cross-validation of protein expression data using mRNA analysis, such as qPCR. Future studies should aim to incorporate qPCR or similar molecular techniques to confirm protein expression findings. We did not have a group with only EGCG-treated rats. The current study focused on whether EGCG can enforce the effect of HA, but for a more detailed analysis of the effect of EGCG and HA, groups with EGCG dispersed in normal saline should be added.

This study analyzed the effects of EGCG-loaded hyaluronic acid hydrogel at a single post-treatment time point (4 weeks). While this provides valuable insights into the efficacy of the treatment, multiple timepoints would have allowed for a more detailed understanding of the healing progression. The study duration was limited to 4 weeks, consistent with the standard timeframe used in similar tendon regeneration research. However, this may not fully capture the durability of the therapeutic effects. Future studies should incorporate longer observation periods to evaluate the sustained efficacy and safety of the treatment, particularly in preparation for clinical applications.

This study utilized only male rats, which may limit the generalizability of the findings to both sexes. Although sex-based differences in tendon regeneration are not commonly evaluated in similar preclinical studies, future research should include both male and female subjects to provide a more comprehensive understanding of potential sex-related variability in therapeutic responses.

While this study mentions the involvement of reactive oxygen species (ROS) pathways in tendon regeneration, specific analyses of ROS pathways were not performed. Future studies should include detailed investigations of these pathways to provide deeper mechanistic insights into the therapeutic effects of EGCG-loaded hyaluronic acid hydrogel. We were unable to conduct drug release experiments for the HA + EGCG compound. However, although we did not directly conduct drug release experiments, previous studies have demonstrated that combining HA with EGCG exhibits an appropriate release mechanism in vivo. Based on the findings reported by Hsiao et al., the release profile of the EGCG-loaded hydrogel exhibited a biphasic release pattern, characterized by an initial burst release of 51.5% within the first 24 h, followed by a sustained release of 40.8% over the subsequent 1–10 days. This resulted in a cumulative release of 92.2% by day 10, which aligns well with the therapeutic requirements for in vivo applications [26]. These results suggest that the EGCG-loaded hydrogel provides an appropriate drug release profile to maintain therapeutic efficacy over the desired timeframe post-injection.

We acknowledge the importance of assessing the stiffness, elasticity, and tensile strength of the treated tendons to better understand their functional recovery. While this study focused on evaluating the biological effects of the treatment, future research could include biomechanical characterization of the tendons to provide a more comprehensive assessment. Additionally, analyzing the mechanical properties of the hydrogel delivery system could further enhance our understanding of its role in tendon healing.

This study did not assess the immunological response to the EGCG-loaded hyaluronic acid hydrogel through an immunohistochemical analysis of immune cell infiltration (e.g., CD68 for macrophages). Such analyses are crucial to understanding the host immune reaction and biocompatibility of the treatment. While this study mentions the involvement of reactive oxygen species (ROS) pathways in tendon regeneration, specific analyses of ROS pathways were not performed. Future studies should include detailed investigations of these fundamental signal pathways to provide deeper mechanistic insights into the therapeutic effects of EGCG-loaded hyaluronic acid hydrogel.

Although further research is needed, these effects suggest that EGCG loaded within hyaluronic acid hydrogel can aid in the healing and restoration of tendon tissue. Through histological and molecular analyses, these hybrid therapeutics were shown to improve the regeneration process of degenerative Achilles tendon in rats. Most importantly, the treatment model in this study has high potential to be used to test innovative therapies that achieve effective treatment outcomes with minimal side effects for patients with degenerative Achilles tendinopathy.

## Figures and Tables

**Table 1:** Composition of experimental groups and collagenase injection dosage, injection, and analysis time points.

Group	Treatment	Dose	Day of Injection	Day of Investigation	Day of Sacrificing
1 (n = 1)	Control	-	-	3	3
2 (n = 1)	Collagenase 30 μL	10 mg/mL	1	3	3
3 (n = 1)	Collagenase 50 μL	10 mg/mL	1	3	3
4 (n = 1)	Collagenase 30 μL	10 mg/mL	1	14	14
5 (n = 1)	Collagenase 50 μL	10 mg/mL	1	14	14
6 (n = 1)	Collagenase 30 μL	3 mg/mL	1	14	14
7 (n = 1)	Collagenase 30 μL	3 mg/mL	1	18	18

## Data Availability

The original contributions presented in this study are included in the article. Further inquiries can be directed to the corresponding author.
